# Tongue Force Training Induces Plasticity of the Lingual Motor Cortex in Young Adult and Aged Rats

**DOI:** 10.3389/fnins.2019.01355

**Published:** 2019-12-19

**Authors:** Miranda J. Cullins, Julie M. Wenninger, Jared S. Cullen, John A. Russell, Jeffrey A. Kleim, Nadine P. Connor

**Affiliations:** ^1^Department of Surgery, University of Wisconsin-Madison, Madison, WI, United States; ^2^School of Biological and Health Systems Engineering, Arizona State University, Tempe, AZ, United States

**Keywords:** tongue, exercise, plasticity, motor cortex, age

## Abstract

Tongue exercise programs are used clinically for dysphagia in aged individuals and have been shown to improve lingual strength. However, the neural mechanisms of age-related decline in swallowing function and its association with lingual strength are not well understood. Using an established rat model of aging and tongue exercise, we hypothesized that the motor cortex of aged rats would have a smaller lingual motor map area than young adult rats and would increase in size as a function of tongue exercise. Over 8 weeks, rats either underwent a progressive resistance tongue exercise program (TE), learned the task but did not exercise (trained controls, TC), or were naïve untrained controls (UC). Cortical motor map areas for tongue and jaw were determined using intracortical microstimulation (ICMS). Rats in the TE and TC groups had a significantly larger motor cortex region for the tongue than the UC group. Lingual cortical motor area was not correlated with protrusive tongue force gains and did not differ significantly with age. These results suggest that learning a novel tongue force skill was sufficient to induce plasticity of the lingual motor cortex yet increasing tongue strength with progressive resistance exercise did not significantly expand the lingual motor area beyond the gains that occurred through the skilled learning component.

## Introduction

Dysphagia is a common condition among aged individuals and is associated with serious health problems, reduced quality of life, and high healthcare costs (Sura et al., 2012; Westmark et al., 2018). Age related changes in swallowing function (presbyphagia), including lingual weakness (Robbins et al., 1995; Hara et al., 2018) and increased swallow durations (Shaw et al., 1995; Nicosia et al., 2000; Namasivayam-MacDonald et al., 2018), may predispose aged individuals to developing dysphagia (Humbert and Robbins, 2008). The mechanisms of age-related decline in swallowing function and its association with lingual strength are not well understood. Determining the underlying causes of declines in oral-motor function with age may lead to more targeted approaches to treat or prevent dysphagia in aged individuals.

An overwhelming body of evidence suggests that CNS changes contribute substantially to sarcopenia, the age-related loss of skeletal muscle mass, strength, and function (Clark and Manini, 2008; Kwan, 2013; Tieland et al., 2018). Age-related reductions in muscle strength and motor control have been associated with motor cortex atrophy and reduced excitability (Clark and Taylor, 2011), but whether such motor cortex changes contribute to declines in lingual motor function are not known. Our previous studies in rats have found age-related changes in neuromuscular junctions in tongue muscles (Hodges et al., 2004; Johnson and Connor, 2011), hypoglossal motor neurons (Schwarz et al., 2009; Behan et al., 2012; Schaser et al., 2012), and the lingual muscles themselves (Ota et al., 2005; Connor et al., 2008; Nagai et al., 2008; Schaser et al., 2011; Cullins and Connor, 2017). In this study we sought to determine whether age is associated with changes in the motor cortex representation of the tongue and jaw. The topographical organization and excitability of the motor cortex can be assessed by using intracortical microstimulation (ICMS) to map cortically driven muscle activation.

Motor cortex plasticity may also be induced by rehabilitative exercises for dysphagia such as tongue exercise. Skilled motor learning has been shown to induce plasticity in the motor cortex, with functional improvements corresponding to increased cortical motor map representation and reduced stimulation thresholds of the involved muscles (Nudo et al., 1996; Kleim et al., 1998; Tennant et al., 2012). Tongue training programs are used clinically for dysphagia in aged individuals and have been shown to improve lingual strength (Robbins et al., 2005; Yeates et al., 2008; Park and Kim, 2016; Rogus-Pulia et al., 2016). There is evidence in young-adult humans and monkeys that tongue-task training induces cortical plasticity (Svensson et al., 2003, 2006; Baad-Hansen et al., 2009; Komoda et al., 2015). However, cortical plasticity may be reduced with age (Seidler et al., 2010; Tennant et al., 2012), and the impact of age on the relationship between tongue training and motor cortex plasticity is not known.

We hypothesized that, compared to young adult rats, the motor cortex of aged rats would have a smaller lingual motor map area and increased lingual motor threshold, assessed by ICMS. We further hypothesized that the cortical movement representation of the tongue would be expanded and movement thresholds reduced as a function of tongue exercise training in young adult and aged rats.

## Materials and Methods

### Animals

This study was performed in compliance with the Guide for Care and Use of Laboratory Animals (8th edition, 2011, National Academies Press) and approved by the Animal Care and Use Committee of the University of Wisconsin School of Medicine and Public Health. Male Fischer 344/Brown Norway rats were obtained from the National Institute on Aging colony (Harlan Laboratories, Indianapolis, IN, United States). Young adult (9 months old at study completion) and old (32 months at study completion) rats were randomly assigned to one of three groups: (1) untrained controls (UC; *n* = 7 young adult, *n* = 8 old) naïve to the tongue force task; (2) trained controls (TC) that learned the tongue force task and performed maximum force testing (*n* = 9 young adult, 9 old), or (3) trained exercise (TE) that underwent the 8-week tongue exercise paradigm described below (*n* = 9 young adult, 9 old). Each group consisted of 10 rats, but final numbers reflect losses due to surgical complications or age-related health concerns. The rats were housed in pairs in standard polycarbonate cages on a 12:12-h light-dark reversed light cycle.

### Tongue Exercise

The clinically based progressive rat tongue exercise training methods have been detailed previously (Connor et al., 2009) and have been shown to increase voluntary tongue force in young and aged rats in a number of studies (Connor et al., 2009; Schaser et al., 2012; Kletzien et al., 2013; Krekeler and Connor, 2017; Cullins et al., 2018). This well-established training paradigm was originally developed based on several studies that used operant conditions in rats to assess licking function (Fowler and Mortell, 1992; Moss et al., 2001; Stanford et al., 2003; Smittkamp et al., 2008). TC and TE rats were water restricted to 3 h/day then trained to press their tongue against an instrumented disk to receive a water reward. Maximum voluntary tongue force was determined at baseline, 4-, and 8-weeks by incrementally increasing the reward force thresholds by 0.002 N after each successful press. This process was repeated over the course of 3 days, and the average of the 10 highest forces was used to determine an individual rat’s maximum voluntary tongue force. The TE group underwent a progressive resistance training program, performed 5 days/week for a total of 8 weeks. Rats exercised at water reward thresholds of 50% max (weeks 1–2), 60% max (weeks 3–4), 70% max (weeks 5–6), and 80% max (weeks 7–8). Force thresholds for weeks 1–4 were calculated from baseline maximums; maximum force testing was repeated in week 4 and the new individual maximums were used to calculate thresholds for weeks 5–8. Each exercise session lasted 10 min. The TC group learned the tongue pressing task and underwent maximum tongue force testing but did not perform progressive tongue exercise. They were placed in the operandum 5 days/week and were allowed to briefly lick water from the disk before they were removed. Body weights were recorded daily to monitor the effects of the water restriction and training paradigms.

### Intracortical Microstimulation (ICMS)

Intracortical Microstimulation was adapted from previously published techniques (Kleim et al., 1998) which have been used to map the orolingual motor area in a rat model of Parkinson disease (Plowman and Kleim, 2011; Plowman et al., 2014). Rats were anesthetized by intraperitoneal (IP) injection (70 mg/kg ketamine and 9 mg/kg xylazine). The plane of anesthesia was monitored for presence of moderate toe-pinch response and maintained either by supplemental IP doses of ketamine (0.1cc as needed) or a femoral i.v. catheter infusion of ketamine (40 mg/kg/hr, adjusted as needed). Anesthesia by infusion was found to be easier to maintain. Anesthesia type (IP or IV) was included as a covariate in the statistical analysis. However, anesthesia type was not found to have any significant influence on map size (*p* = 0.554; combined jaw and tongue area ±SD: IP = 1.91 ± 1.04 mm^2^, *N* = 26; IV = 2.18 ± 0.83 mm^2^, *N* = 25). Animals were placed in a stereotaxic apparatus and the skull and dura were removed from 5 to 6 mm rostral and 2–3 mm caudal to Bregma, over the left motor cortex; up to 5 mm lateral. A small puncture was made in the cisterna magna prior to removing the skull and dura to prevent edema. Warm silicone oil was placed on the cortical surface. A digital image of the cortical surface was taken and a 250 μm grid was superimposed onto the image and used as a guide to select and track stimulation sites. A glass microelectrode filled with 3.5 M saline solution and a platinum wire (600–800 kΩ) controlled by a hydraulic microdrive was used to make systematic penetrations across the cortex using the cortical surface image and grid as a guide. At each penetration site, the electrode was lowered to approximately 1550 μm from the surface to correspond with cortical layer V. Stimulation consisted of thirteen 200 μs cathodal pulses delivered at 350 Hz from an electrically isolated stimulation circuit. Rats were maintained in a prone position with the forelimb consistently supported. At each site the stimulating current was gradually increased until a movement was detected, up to 60 μA. Once movement was detected, the stimulating current was gradually lowered until the movement disappeared then increased again to establish the movement threshold at which a motor response occurred 6 of 10 stimulations. If more than one muscle group was activated at the threshold current, that site was considered positive for both and included in the map size calculation for each (ex: jaw + tongue or jaw + neck). If no movement could be detected at 60 μA, the site was determined to be non-responsive. The initial penetration site was randomly selected. Once movement of any sort (including elbow or neck) was established at the initial penetration, sites likely to border tongue or jaw motor areas were targeted. The jaw and tongue map areas are typically bordered rostrally and laterally by non-responsive sites, and caudally and medially by sites activating the vibrissae, neck, and forelimb (Neafsey et al., 1986). The next penetration site, regardless of response, was selected at least 2 grid lengths from the previous activation site whenever possible to avoid temporary threshold changes due to recent adjacent stimulation. Movements of the tongue and jaw were differentiated visually and confirmed as needed by accelerometers attached to the tongue and jaw.

The procedure was continued until the entire jaw and tongue representations were mapped, as both the tongue and jaw muscles contribute to swallowing and may be activated by the tongue force task. Representation area was calculated by multiplying the number of stimulation sites by 0.0625 mm^2^.

### Statistical Analysis

Motor cortical representations of the tongue and jaw were assessed by MANCOVA. Independent variables were age and treatment (UC, TC, TE), and dependent variables were tongue and jaw area. Anesthesia type (IP or IV) and weight were included as covariates. Rats grow throughout their lifespan, so old rats typically weigh more than young rats (Turturro et al., 1999). Weight was included as a covariate based on previous studies that found body weight and brain volume to be correlated in the rat (Bailey et al., 2004; Oguz et al., 2013; Welniak-Kaminska et al., 2019). Over the course of the 8-week training paradigm old rats lost on average 7% of their body weight and young rats gained 12%. Mapping occurred at the end of the study and weight at the time of mapping was used. Estimated marginal means and standard errors are reported with the covariate body weight at 575 g. Movement thresholds of the tongue and jaw were also assessed by MANCOVA. Independent variables were age and treatment (UC, TC, TE), dependent variables were mean tongue and jaw thresholds (μA), and anesthesia type (IP or IV) was included as a covariate. The Pillai’s trace test statistics are reported. MANCOVA was followed by individual univariate ANCOVAs.

A 2-way and repeated measures ANOVA were used to assess change in maximum tongue force from baseline to 8 weeks by age and treatment group with weight included as a covariate. A Pearson correlation was used to assess the relationship between tongue motor representation area and change in maximum tongue force.

## Results

### Tongue Cortical Area

**FIGURE 1:**
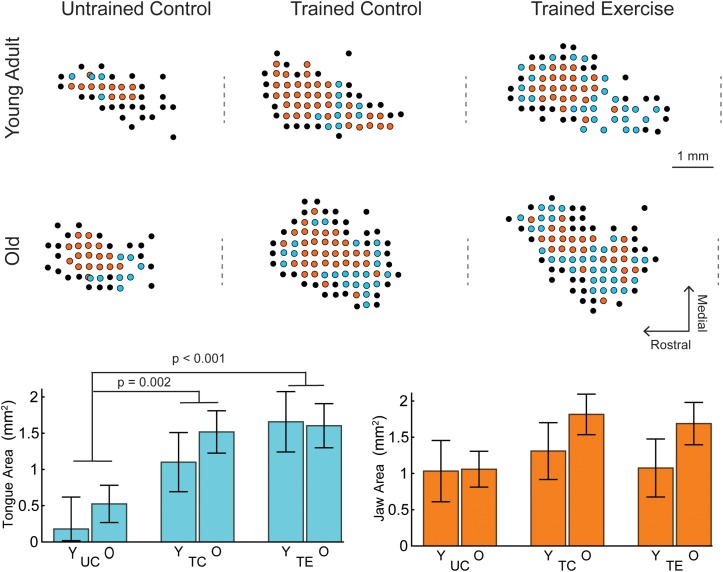
Examples of tongue and jaw representations of the left motor cortex determined by ICMS, selected to represent mean group values. Each dot represents an ICMS site colored by motor response (Cyan, tongue; Orange, jaw; Black, other/no response). The craniotomy over the left motor cortex ranged from +6 to –2 AP from Bregma. Positive jaw and tongue sites were typically within +5 to +2 AP. The approximate AP location of Bregma is indicated by a dashed line to the left of each map. **Lower Left:** For all ages (Y, young adult rats; O, old rats), tongue area in the trained exercise (TE) and trained control (TC) groups was significantly larger than the naïve untrained control group (UC). There were no significant differences in tongue area with age. **Lower Right:** There were no significant differences with age or tongue exercise for jaw area. Tongue and jaw map areas represented in bar charts are the estimated marginal means ±standard error.

Examples of tongue and jaw representations in the motor cortex determined by ICMS are shown in Figure 1. MANCOVA results indicated a significant main effect of treatment group (UC, TC, TE) across the dependent variables of Tongue and Jaw motor cortical area [Pillai’s Trace = 0.473, *F*(4,86) = 6.668, *p* < 0.001, η_p_^2^ = 0.237]. There was not a significant main effect for age [Pillai’s Trace = 0.104, *F*(2,42) = 2.43, *p* = 0.101], or an age-treatment interaction [Pillai’s Trace = 0.065, *F*(4,86) = 0.725, *p* = 0.577].

The MANCOVA treatment results were followed up with univariate ANCOVA tests which indicated a significant treatment effect for tongue area [*F*(2,43) = 9.537, *p* < 0.001, η_p_^2^ = 0.307] but not jaw area [*F*(2,43) = 1.680, *p* = 0.1.98, η_p_^2^ = 0.072]. Pairwise comparisons indicated that the area of tongue motor cortical representation was significantly larger in each of the trained groups (TC = 1.31 mm^2^ ± 0.31 SE, TE = 1.63 mm^2^ ± 0.32 SE) compared to the untrained control group (0.352 mm^2^ ± 0.27 SE; *p* = 0.002 and *p* < 0.001 respectively; estimated marginal means reported at weight = 575 g). The mean tongue area of the exercise group was not significantly larger than the trained control group (*p* = 0.143).

### Movement Thresholds

**FIGURE 2:**
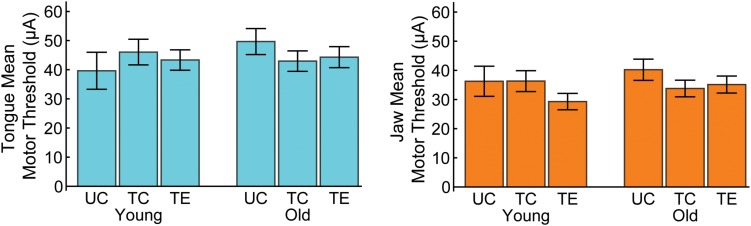
No significant differences were found in tongue or jaw motor thresholds with age or tongue exercise.

The effects of age and treatment on movement thresholds, the mean minimum stimulation current that elicited jaw or tongue movement, was evaluated in a separate MANCOVA with anesthesia (IP or IV) as a covariate. No significant age [Pillai’s Trace = 0.027, *F*(2,35) = 0.475, *p* < 0.63, η_p_^2^ = 0.026], treatment [Pillai’s Trace = 0.068, *F*(4,72) = 0.64, *p* = 0.64, η_p_^2^ = 0.034], or interaction effects [Pillai’s Trace = 0.10, *F*(4,72) = 0.95, *p* = 0.44, η_p_^2^ = 0.05] were found for tongue and jaw motor thresholds (Figure 2).

### Tongue Force

A 2-way ANOVA was used to determine the effects of age and exercise (TE, TC) on the change in maximum tongue force (8 weeks – baseline). The main effect of exercise was significant *F*(1,31) = 72.76, *p* < 0.001, η_p_^2^ = 0.701; the change in maximum tongue force was significantly greater after 8 weeks of tongue exercise (TE group) compared to the trained control (TC) group (77.66 mN ± 5.43 SE, 12.11 mN ± 5.43 SE). Age and age-treatment interaction effects were not significant [*F*(1,31) = 0.889, *p* = 0.353, η_p_^2^ = 0.028, *F*(1,31) = 3.54, *p* = 0.069, η_p_^2^ = 0.102].

**FIGURE 3:**
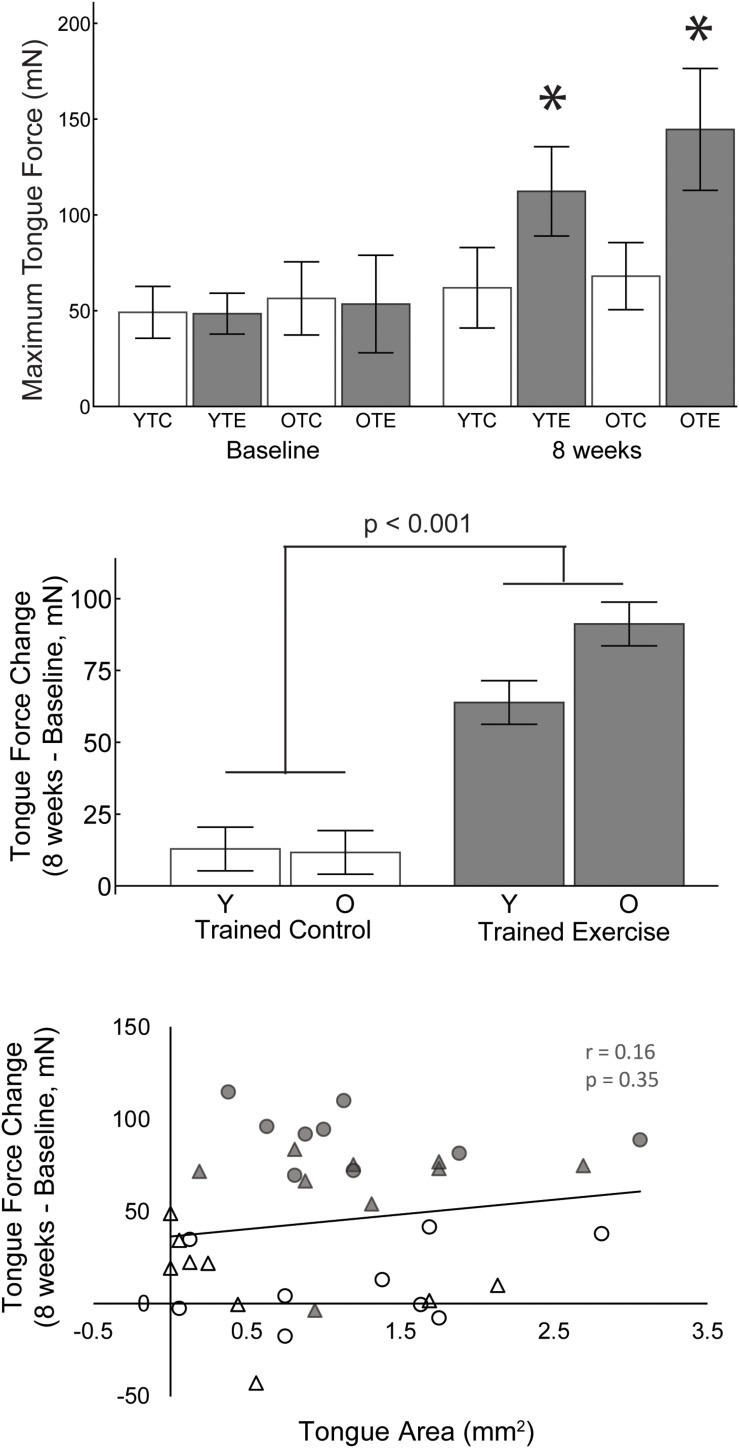
**Upper:** Maximum tongue force values for both age groups, trained exercise and trained control, at baseline and 8 weeks. Asterisks indicate significant difference from baseline and from age matched control groups at 8 weeks (*p* < 0.001). **Middle:** The change in maximum tongue force from baseline was significantly greater after 8 weeks of tongue exercise compared to the trained control group. **Lower:** The area of tongue motor representation was not significantly correlated with the change in tongue force. White, trained control; Gray, trained exercise; Circles, old; triangles, young adult.

Tongue force was not significantly correlated with tongue motor area (Figure 3; *r* = 0.16, *p* = 0.35) and variance in the cortical map size accounted for very little variance (<3%, *r*^2^ = 0.026) in increased tongue force.

## Discussion

This study found that rats that performed protrusive tongue force training and testing, with or without progressive resistance exercise, had a significantly larger motor cortex region representing the tongue than the untrained control group. No significant differences with age or changes in motor threshold were found. A previous study that assessed total cortical orolingual movement area (jaw, tongue, and lips combined) in the rat after shorter tongue force training protocol (4–6 day), in contrast to our results, found a decreased motor threshold, but no change in motor area (Guggenmos et al., 2009). Motor area expansions can occur at the expense of the area of adjacent muscle groups (Kleim et al., 1998), thus motor area changes due to tongue training may not be detected when measuring the total oral-lingual motor area. We found that tongue training specifically increased tongue representation, though we did not find a significant reduction in jaw area (Figure 1). Additional contributing factors may include differences in the training paradigms and timing of motor cortex assessment.

### Skilled Motor Learning vs. Strength

Studies of skilled, unskilled, and strength-based forearm reaching tasks in rats have found that only skilled motor learning induces plasticity of the motor cortex, in which case increased area is associated with motor gains (Kleim et al., 1998; Remple et al., 2001). Our study is in agreement with these results in that both groups that learned the tongue force task (trained control and trained exercise) had an increased area of tongue representation in the motor cortex, but the exercise group did not have a significantly larger tongue area than the trained control group (Figure 1). It is worth noting that the trained control group, which learned the task and underwent maximum force testing, experienced a small yet significant increase in maximum tongue force from baseline (Figure 3). Furthermore, tongue area was not predictive of maximum tongue force (Figure 3). Plasticity contributing to lingual force gains may be encoded outside of the motor cortex; some studies have suggested that plasticity relevant to limb strength training occurs in the spinal cord (Sale et al., 1983; Griffin and Cafarelli, 2005). Similarly, a previous study from our lab found that the tongue training paradigm increased neuroplasticity associated neurotrophins in the tongue motor nuclei of the brainstem (Schaser et al., 2012, 2016).

### Movement Thresholds

We hypothesized the aged group would have increased movement thresholds because age has been associated with reduced excitability of the motor cortex (Bhandari et al., 2016). However, we did not find any significant differences in the average threshold (minimum stimulation current) that induced tongue or jaw movement (Figure 2). We also did not find a significant change in threshold with tongue training. Reports on motor training threshold changes with ICMS have been mixed (Remple et al., 2001; Guggenmos et al., 2009) and it has been suggested that motor threshold decreases occur after the map area has returned to baseline (Tennant et al., 2012). This may explain why we did not find a change in threshold with training, as the tongue motor map area was still significantly expanded at the end of the study.

### Motor Plasticity With Age

We hypothesized that plasticity of the motor cortex would be reduced with age, but our results did not support this hypothesis. A previous study using ICMS and skilled reaching reported that aged animals can learn new motor tasks, but do so without motor map expansions (Tennant et al., 2012). In our study the difference in tongue motor area between the trained exercise and untrained groups appears smaller in the aged vs. young rats (Figure 1), but these differences were not significant. Neuroplasticity in aged individuals has also been described as a critical mechanism of preserving motor function, including mastication, by recruiting additional brain regions beyond the sensorimotor areas to compensate for declining peripheral and CNS tissues (Clark and Taylor, 2011; Avivi-Arber and Sessle, 2018). Future studies could determine whether tongue exercise training also induces plasticity in the functional connectivity of brain regions activated during swallowing.

### Timing

Previous studies on skilled reaching have suggested that reorganization of cortical motor maps may be transient, and return to baseline after the initial learning period, despite maintaining the skill (Molina-Luna et al., 2008; Tennant et al., 2012). Our results appear to differ as we found significantly increased map size after 8 weeks of training. This difference may be due to the tongue training task being progressive over the course of training. In the previous studies of skilled reaching, the majority of performance gains occurred the first week of training. In the current study, the tongue exercise reward threshold was increased every other week and terminated with 3 days of peak voluntary force testing in both training groups. Thus, our results suggest that if the motor task continues to be challenging and produce performance gains, the motor map expansion may continue over a longer time frame. Additionally, the current study investigates the corticobulbar rather than corticospinal system, and the pattern of experience dependent plasticity may differ between these two systems.

### Limitations

This study established that a clinically based lingual exercise program is sufficient to induce plasticity of the motor cortex, but no assessments of swallowing function were included. Thus, we cannot address whether the lingual motor cortex plasticity induced by tongue training transfers any benefits to swallowing function. This will be an important topic to address in future studies as clinical tongue strengthening trials have shown improvements in swallowing related outcomes (Rogus-Pulia et al., 2016), but the efficacy of tongue exercise as a treatment for dysphagia is not well established (Langmore and Pisegna, 2015). Dysphagia etiology may also be a key factor in establishing the biological mechanisms by which tongue exercise may improve swallowing function. Therefore, it may be advisable to address these questions of plasticity specificity and transference in specific disease models with established swallowing deficits such as Parkinson’s (Russell et al., 2013) and stroke (Cullins and Connor, 2019).

We did not find any significant differences in jaw and tongue motor area with age, but this comparison may have been limited by two different factors. First, we assessed cortical area by stimulating at a constant superficial cortical depth within layer V of the M1 motor region. However, other studies have reported ICMS can evoke tongue and jaw movements from multiple depths within layers 5 and 6 of M1, the adjacent S1 region, and the gustatory insular cortex (Neafsey et al., 1986; Adachi et al., 2007; Avivi-Arber et al., 2010a, b, 2015). Additionally, cortical thinning may occur with age (Salat et al., 2004). Thus, more accurate orolingual motor maps and aging comparisons may require measuring the total 3D cortical volume that can elicit movements of tongue and jaw. In addition to mapping deeper sites, lingual EMG recordings may be used to improve detection of sites that evoke more subtle twitches of the tongue (Adachi et al., 2007; Avivi-Arber et al., 2010a, b). An additional limitation in our age group comparison was that rats continue to grow with age thus the aged group weighed more than the young adult group. Weight was included as a covariate because brain volume may increase with body weight, but this may still be a confounding factor for age group comparisons.

### Conclusion

We found that tongue training induced plasticity of the motor cortex in both young and aged rats, but further studies are needed to establish the relevance of lingual motor plasticity for dysphagia rehabilitation.

## Data Availability Statement

The datasets generated for this study are available on request to the corresponding author.

## Ethics Statement

The animal study was reviewed and approved by the Animal Care and Use Committee of the University of Wisconsin, School of Medicine and Public Health.

## Author Contributions

NC and JK designed and supervised the study. JW, JR, JC, and MC collected, analyzed, and interpreted the data. MC wrote the manuscript with input from all authors. All authors approved the manuscript.

## Conflict of Interest

The authors declare that the research was conducted in the absence of any commercial or financial relationships that could be construed as a potential conflict of interest.
